# Melatonin ameliorates myocardial ischemia/reperfusion injury in type 1 diabetic rats by preserving mitochondrial function: role of AMPK-PGC-1α-SIRT3 signaling

**DOI:** 10.1038/srep41337

**Published:** 2017-01-25

**Authors:** Liming Yu, Bing Gong, Weixun Duan, Chongxi Fan, Jian Zhang, Zhi Li, Xiaodong Xue, Yinli Xu, Dandan Meng, Buying Li, Meng Zhang, Bin Zhang, Zhenxiao Jin, Shiqiang Yu, Yang Yang, Huishan Wang

**Affiliations:** 1 Department of Cardiovascular Surgery, General Hospital of Shenyang Military Area Command, 83 Wenhua Road, Shenyang, Liaoning 110016, China; 2 Department of Cardiovascular Surgery, Xijing Hospital, The Fourth Military Medical University, 127 Changle West Road, Xi’an 710032, China; 3 Department of Thoracic and Cardiovascular Surgery, Affiliated Drum Tower Hospital of Nanjing University Medical School, 321 Zhongshan Road, Nanjing, Jiangsu 210008, China; 4 Department of Thoracic Surgery, Tangdu Hospital, The Fourth Military Medical University, 1 Xinsi Road, Xi’an, Shaanxi 710032, China; 5 Department of Natural Medicine, School of Pharmacy, The Fourth Military Medical University, 169 Changle West Road, Xi’an 710032, China; 6 Department of Biomedical Engineering, The Fourth Military Medical University, 169 Changle West Road, Xi’an 710032, China

## Abstract

Enhancing mitochondrial biogenesis and reducing mitochondrial oxidative stress have emerged as crucial therapeutic strategies to ameliorate diabetic myocardial ischemia/reperfusion (MI/R) injury. Melatonin has been reported to be a safe and potent cardioprotective agent. However, its role on mitochondrial biogenesis or reactive oxygen species (ROS) production in type 1 diabetic myocardium and the underlying mechanisms remain unknown. We hypothesize that melatonin ameliorates MI/R injury in type 1 diabetic rats by preserving mitochondrial function via AMPK-PGC-1α-SIRT3 signaling pathway. Both our *in vivo* and *in vitro* data showed that melatonin reduced MI/R injury by improving cardiac function, enhancing mitochondrial SOD activity, ATP production and oxidative phosphorylation complex (II, III and IV), reducing myocardial apoptosis and mitochondrial MDA, H_2_O_2_ generation. Importantly, melatonin also activated AMPK-PGC-1α-SIRT3 signaling and increased SOD2, NRF1 and TFAM expressions. However, these effects were abolished by Compound C (a specific AMPK signaling blocker) administration. Additionally, our cellular experiment showed that SIRT3 siRNA inhibited the cytoprotective effect of melatonin without affecting p-AMPK/AMPK ratio and PGC-1α expression. Taken together, we concluded that melatonin preserves mitochondrial function by reducing mitochondrial oxidative stress and enhancing its biogenesis, thus ameliorating MI/R injury in type 1 diabetic state. AMPK-PGC1α-SIRT3 axis plays an essential role in this process.

Ischemic heart disease (IHD) is a leading cause of death in type 1 diabetic patients worldwide^1^,^2^. Although timely reperfusion is the optimal therapeutic strategy, reperfusion itself can result in lethal cardiac damage. Previously, we and others reported that diabetes aggravated myocardial ischemia/reperfusion (MI/R) injury although the underlying mechanisms remain largely unknown^3^,^4^. Notably, mitochondrial dysfunction has been recognized as a critical contributor to the poor prognosis of IHD in diabetic setting. On one hand, diabetes impairs myocardial mitochondrial biogenesis, leading to loss of mitochondrial number and function, which eventually causes cardiac contractile dysfunction^5^,^6^. On the other hand, as mitochondria is the major source of reactive oxygen species (ROS) in the heart, the prolonged hyperglycemia in diabetic state significantly increases mitochondrial ROS generation by disturbing the balance of peroxidases such as selenium glutathione peroxidase and xanthine dehydrogenase^7^. This ultimately aggravates the apoptosis and necrosis of cardiomyocytes. To this end, enhancing mitochondrial biogenesis and reducing mitochondrial oxidative stress have emerged as crucial therapeutic strategies to ameliorate diabetic MI/R injury^8^.

Melatonin (N-acetyl-5-methoxytryptamine) is deemed as a powerful endogenous antioxidant due to its direct free-radical scavenging activity and indirect anti-oxidative property^9^,^10^. Importantly, more and more studies have indicated its cardioprotective actions. Of interest, we previously demonstrated that melatonin exerted a solid protective effect against MI/R injury in type 2 diabetic rats^4^. At the same time, melatonin has been found to preserving mitochondrial function in diabetic state in multiple organs^11^,^12^,^13^,^14^. However, whether melatonin regulate mitochondrial biogenesis or ROS production in type 1 diabetic myocardium and the underlying mechanisms remain unknown.

AMP-activated protein kinase (AMPK) is a crucial intracellular serine/threonine protein kinase which functions as a fuel sensor in the heart^15^. AMPK-activated peroxisome proliferator-activated receptor (PPARγ) coactivator-1α (PGC-1α) has been demonstrated to play a key role in the regulation of mitochondrial biogenesis and oxidative stress^16^. Moreover, silent mating-type information regulation 2 homolog 3 (SIRT3) has been found to serve as the downstream target of AMPK-PGC-1α signaling, which enhances mitochondrial biogenesis and the deacetylation of mitochondrial anti-oxidative enzymes^17^. Growing evidence reported that SIRT3 activation prevented myocardial mitochondrial oxidative stress and damage in multiple pathological conditions^18^,^19^,^20^. Additionally, a recent study by Pi *et al*. also demonstrated that melatonin could activate hepatic SIRT3 signaling, thus reducing cadmium-induced hepatotoxicity^21^. However, whether melatonin modulate myocardial SIRT3 and its up and downstream regulatory signalings are still unknown.

On the basis of the above observations, *in vivo* and *in vitro* studies were designed to: (1) investigate whether melatonin enhances mitochondrial biogenesis and preserves mitochondrial function, thus reducing MI/R injury in type 1 diabetic state; (2) determine the potential roles of AMPK-PGC-1α signaling and SIRT3 signaling in melatonin’s cardioprotective actions.

## Results

### Streptozotocin injection induced type 1 diabetic rats exhibited impaired glucose tolerance and reduced myocardial AMPK/PGC-1α and SIRT3 signaling

**Figure 1:**
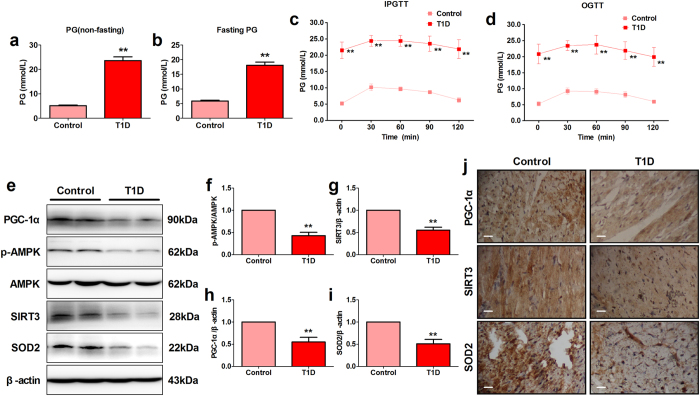
Streptozotocin injection induced type 1 diabetic rats exhibited impaired glucose tolerance and reduced myocardial AMPK/PGC-1α and SIRT3 signaling. (**a**) Non-fasting plasma glucose of control rats and type 1 diabetic rats after 7 days of streptozotocin injection. (**b**) Fasting plasma glucose of control rats and type 1 diabetic rats after 7 days of streptozotocin injection. (**c**) Intraperitoneal glucose tolerance test after 7 days of streptozotocin injection. (**d**) Oral glucose tolerance test (OGTT) after 7 days of streptozotocin injection. (**e**) Representative blots. Myocardial p-AMPK, PGC-1α, SIRT3 and SOD2 expressions were evaluated after 1 month of streptozotocin injection. (**f**) p-AMPK/AMPK ratio. (**g**) SIRT3 expression. (**h**) PGC-1α expression. (**i**) SOD2 expression. (**j**) Representative myocardial immunohistochemistry images of PGC-1α, SIRT3 and SOD2 (120×, bar = 400 μm). The depicted data are the means ± SEM, n = 6/group. ^**^P < 0.01 vs. the Control group. T1D, type 1 diabetes. PG, plasma glucose. IPGTT, intraperitoneal glucose tolerance test. OGTT, oral glucose tolerance test.

To confirm the type 1 diabetic animal model was established successfully, we measured the non-fasting and fasting plasma glucose levels after 7 days of streptozotocin (STZ) injection. As shown in Fig. 1a,b, STZ-injected rats exhibited significantly increased non-fasting and fasting plasma glucose levels (*P* < 0.01, compared with the Control group). Those with fasting plasma glucose above 11.1 mmol/l were considered as diabetic. We next performed IPGTT and OGTT to further determine whether the glucose tolerance was significantly impaired in STZ-injected rats. As expected, type 1 diabetic rats showed markedly impaired IPGTT and OGTT (Fig. 1c,d). Interestingly, further western blot data and immunohistochemistry staining showed that type 1 diabetes markedly down-regulated myocardial AMPK phosphorylation level and the expressions of PGC-1α, SIRT3 and SOD2 (Fig. 1e–j).

### Compound C abolished melatonin-induced cardioprotective effect on myocardial ischemia/reperfusion injury in type 1 diabetic rats

**Figure 2:**
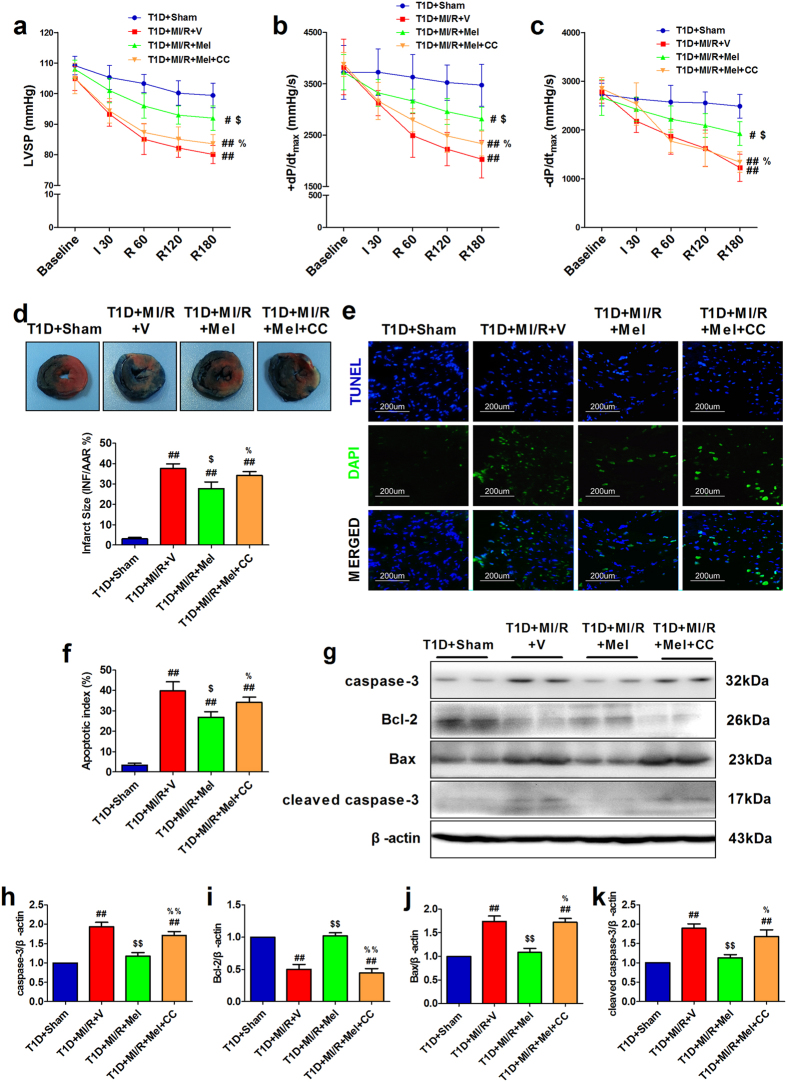
Compound C abolished melatonin-induced cardioprotective effect on myocardial ischemia/reperfusion injury in type 1 diabetic rats. Myocardial ischemia/reperfusion surgery was performed after 1 month of streptozotocin injection. (**a**) Left ventricular systolic pressure. (**b**) and (**c**) The first derivative of left ventricular pressure (+dP/dt_max_ and −dP/dt_max_). Cardiac functional data was continuously monitored during the ischemia (30 min) and reperfusion period (3 hours). (**d**) Myocardial infarct size. (**e**) *In situ* detection of apoptotic cardiomyocytes by TUNEL staining (200×). (**f**) Myocardial apoptotic index. (**g**) Representative blots. (**h**) caspase-3 expression. (**i**) Bcl-2 expression. (**j**) Bax expression. (**k**) Cleaved caspase-3 expression. The depicted data are the means ± SEM, n = 6/group. ^##^*P* < 0.01/^#^*P* < 0.05 vs the T1D + Sham group, ^$$^*P* < 0.01/^$^*P* < 0.05 vs the T1D + MI/R + V group, ^%%^*P* < 0.01/^%^*P* < 0.05 vs the T1D + MI/R + Mel group. T1D, type 1 diabetes. Mel, melatonin. CC, Compound C. V, vehicle. LVSP, left ventricular systolic pressure.

To determine the effect of melatonin on ischemia/reperfusion injury in type 1 diabetic setting and the underlying mechanisms, we employed Compound C (a specific AMPK signaling blocker) in our *in vivo* experiment. No significant changes in cardiac function and apoptotic signaling were found between T1D + MI/R + V group and T1D + MI/R + CC group (Supplementary Fig. S2a–f), indicating that under experimental dosage, Compound C caused no significant effects on diabetic heart. After 3 hours of reperfusion, we found that melatonin treatment significantly improved LVSP and ±dP/dt_max_ (Fig. 2a–c, *P* < 0.05, compared with the T1D + MI/R + V group). Meanwhile, melatonin-treated group also exhibited reduced myocardial infarction and apoptotic index (Fig. 2d–f, *P* < 0.05, compared with the T1D + MI/R + V group). However, these protective actions were abolished by Compound C administration (Fig. 2a–f, *P* < 0.05, compared with the T1D + MI/R + Mel group). Additionally, melatonin also significantly inhibited apoptotic signaling pathway by reducing the expressions of caspase-3, bax and cleaved caspase-3 and increasing Bcl-2 level (Fig. 2g–k, *P* < 0.05, compared with the T1D + MI/R + V group). These effects were also inhibited by Compound C (Fig. 2g–k, *P* < 0.05, compared with the T1D + MI/R + Mel group). These data all indicated that AMPK signaling played a pivotal role in melatonin’s cardioprotective effect.

### Compound C impaired mitochondrial function and blunted melatonin-induced suppression on mitochondrial oxidative damage in diabetic myocardium

**Figure 3:**
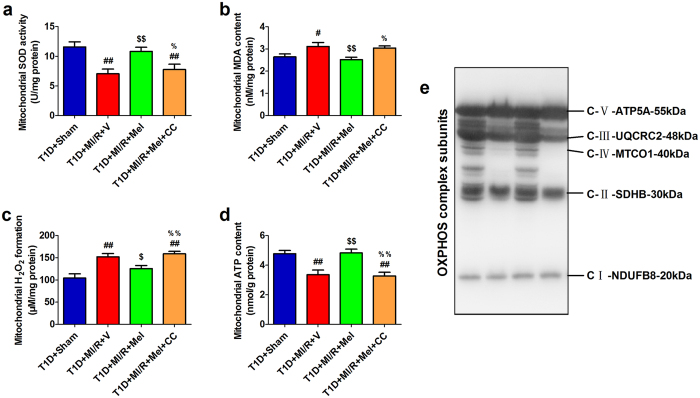
Compound C impaired mitochondrial function and blunted melatonin-induced suppression on mitochondrial oxidative damage in diabetic myocardium. The mitochondrial oxidative stress markers and OXPHOS complex subunits expression were detected after 3 hours of reperfusion. (**a**) Mitochondrial SOD activity. (**b**) Mitochondrial MDA content. (**c**) Mitochondrial H_2_O_2_ formation. (**d**) Mitochondrial ATP content. (**e**) Representative blots of mitochondrial OXPHOS complex subunits. The depicted data are the means ± SEM, n = 6/group. ^##^*P* < 0.01/^#^*P* < 0.05 vs the T1D + Sham group, ^$$^*P* < 0.01/^$^*P* < 0.05 vs the T1D + MI/R + V group, ^%%^*P* < 0.01/^%^*P* < 0.05 vs the T1D + MI/R + Mel group. T1D, type 1 diabetes. Mel, melatonin. CC, Compound C. V, vehicle.

To assess the mitochondrial function in this setting, we evaluated mitochondrial SOD activity, MDA content, H_2_O_2_ formation and ATP content in these experimental groups. As shown in Fig. 3a–d, melatonin effectively preserved mitochondrial function by improving mitochondrial SOD activity and ATP production, and reducing mitochondrial MDA and H_2_O_2_ generation (*P* < 0.05, compared with the T1D + MI/R + V group). Interestingly, inhibition of AMPK signaling also blunted these effects and impaired mitochondrial function (*P* < 0.05, compared with the T1D + MI/R + Mel group). Furthermore, we assessed the expressions of mitochondrial electron transport chain complexes using western blot analysis. In agreement with the previous data, we found that oxidative phosphorylation (OXPHOS) complex subunits (complexes II, III and IV) were up-regulated in melatonin-treated group (Fig. 3e, compared with the T1D + MI/R + V group). However, Compound C administration also blocked this effect (Fig. 3e, compared with the T1D + MI/R + Mel group).

### Compound C inhibited myocardial AMPK/PGC-1α signaling in diabetic myocardium

**Figure 4:**
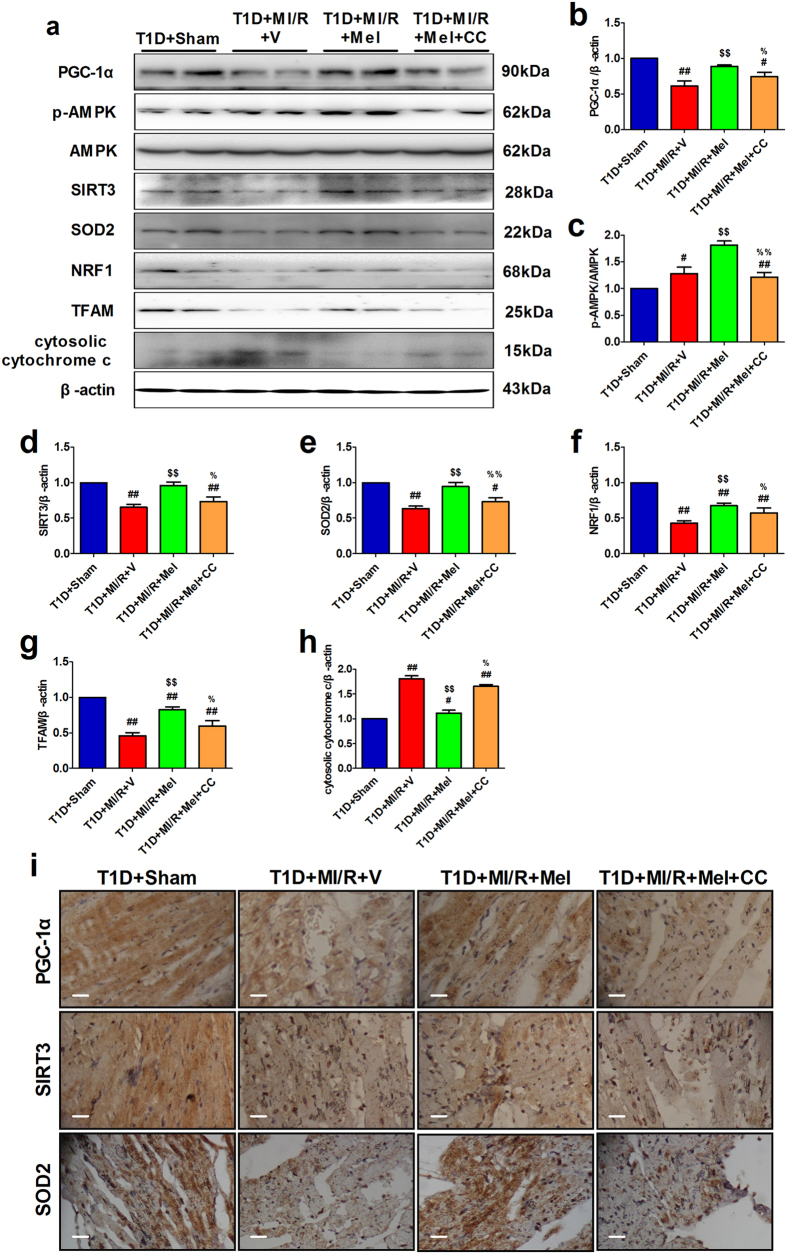
Compound C inhibited myocardial AMPK/PGC-1α signaling in in diabetic myocardium. The western blot analysis was performed after 3 hours of reperfusion. (**a**) Representative blots. (**b**) PGC-1α expression. (**c**) p-AMPK/AMPK ratio. (**d**) SIRT3 expression. (**e**) SOD2 expression. (**f**) NRF1 expression. (**g**) TFAM expression. (**h**) Cytosolic cytochrome c expression. (**i**) Representative myocardial immunohistochemistry images of PGC-1α, SIRT3 and SOD2 (120×, bar = 400 μm). The depicted data are the means ± SEM, n = 6/group. ^##^*P* < 0.01/^#^*P* < 0.05 vs the T1D = Sham group, ^$$^*P* < 0.01/^$^*P* < 0.05 vs the T1D = MI/R = V group, ^%%^*P* < 0.01/^%^*P* < 0.05 vs the T1D = MI/R = Mel group. T1D, type 1 diabetes. Mel, melatonin. CC, Compound C. V, vehicle.

After 3 hours of reperfusion, melatonin significantly increased the expressions of PGC-1α, SIRT3, SOD2, NRF1, TFAM and the p-AMPK/AMPK ratio, decreased cytosolic cytochrome c level (Fig. 4a–h, *P* < 0.05, compared with the T1D + MI/R + V group). However, these effects were also abolished by Compound C administration (Fig. 4a–h, *P* < 0.05, compared with the T1D + MI/R + Mel group). We next carried out immunohistochemistry analysis. Consistently, we found that melatonin-treated group also showed increased PGC-1α, SIRT3 and SOD2 staining, while inhibition of AMPK by Compound C blunted these effects (Fig. 4i).

### Compound C and SIRT3 siRNA transfection blunted melatonin-induced anti-apoptotic effect against SIR injury in high glucose medium treated H9c2 cells

**Figure 5:**
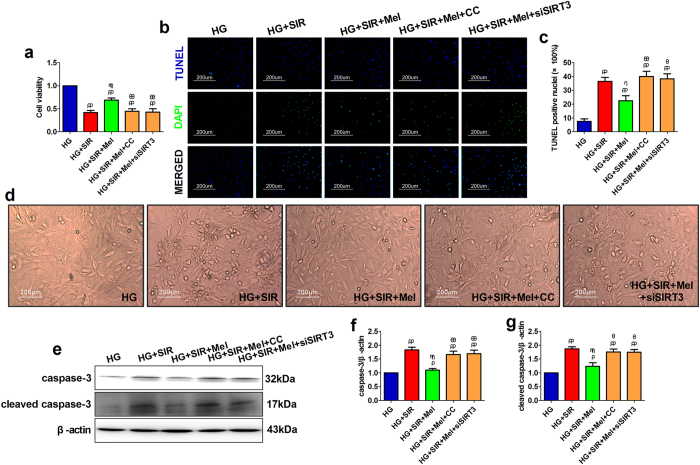
Compound C and SIRT3 siRNA transfection blunted melatonin-induced anti-apoptotic effect against SIR injury in high glucose medium treated H9c2 cells. The H9c2 cells were exposed to high glucose medium (33 mmol/l) for 6 hours before the SIR treatment and during the entire reperfusion period (4 hours). HG-treatment was co-administered with or without melatonin (10 μmol/l) to evaluate its cytoprotective effect. Compound C (3 μmol/l) was administered for 6 hours before the SIR exposure to inhibit the AMPK signaling. (**a**) Cellular viability was presented by dividing the optical density of samples with that of the HG group. (**b**) Representative images of TUNEL staining (200×). (**c**) Percentage of TUNEL positive nuclei. (**d**) Cellular morphology (200×). (**e**) Representative blots. (**f**) Caspase-3 expression. (**g**) Cleaved caspase-3 expression. The depicted data are the means ± SEM, n = 6/group. ^ρρ^*P* < 0.01/^ρ^*P* < 0.05 vs the HG group, ^ηη^*P* < 0.01/^η^*P* < 0.05 vs the HG + SIR group, ^θθ^*P* < 0.01/^θ^*P* < 0.05 vs the HG + SIR + Mel group. HG, high glucose. SIR, simulated ischemia reperfusion. Mel, melatonin. CC, Compound C.

To further confirm the cardioprotective mechanisms of melatonin in promoting mitochondrial function in type 1 diabetic heart, we performed *in vitro* experiment using H9c2 cardiomyoblasts. Initially, we found that 6 hours of high glucose incubation reduced AMPK phosphorylation and the expressions of PGC-1α, SIRT3 and SOD2 in a glucose concentration-dependent manner (Supplementary Fig. S1, *P* < 0.05, compared with the NG+mannitol group). Then, we evaluated the transfection efficiency of SIRT3 small interfering RNA. As shown in Supplementary Fig. S2g, compared with the SIR+control siRNA group, SIRT3 siRNA significantly down-regulated SIRT3 expression (*P* < 0.05). Melatonin not only improved cell viability but also reduced cellular apoptosis by decreasing the percentage of TUNEL positive nuclei and inhibiting the expressions of caspase-3 and cleaved caspase-3 (Fig. 5a–c,e–g, *P* < 0.05, compared with the HG + SIR group). However, these cytoprotective effect were blunted by Compound C or SIRT3 siRNA treatment (Fig. 5a–c,e–g, *P* < 0.05, compared with the HG + SIR + Mel group). Consistently, melatonin effectively alleviated cellular shrinkage and detachment induced by SIR treatment, while this effect was also inhibited by Compound C or SIRT3 siRNA (Fig. 5d), indicating that AMPK signaling and SIRT3 signaling are both key signaling pathways that mediate melatonin’s protective actions.

### Compound C and SIRT3 siRNA transfection impaired mitochondrial function and inhibited melatonin-induced suppression on mitochondrial oxidative damage in high glucose medium treated H9c2 cells

**Figure 6:**
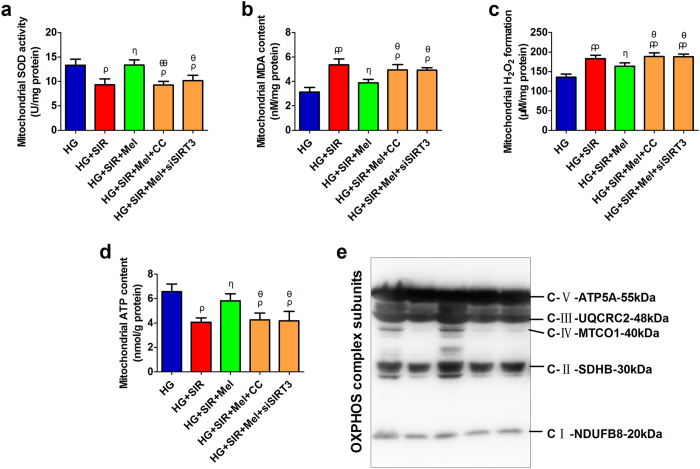
Compound C and SIRT3 siRNA transfection impaired mitochondrial function and inhibited melatonin-induced suppression on mitochondrial oxidative damage in high glucose medium treated H9c2 cells. H9c2 cells were incubated in high glucose medium (33 mmol/l) for 6 hours before the SIR treatment and during the entire reperfusion period (4 hours) with or without the treatment of melatonin (10 μmol/l) or Compound C (3 μmol/l). (**a**) Mitochondrial SOD activity. (**b**) Mitochondrial MDA content. (**c**) Mitochondrial H_2_O_2_ formation. (**d**) Mitochondrial ATP content. (**e**) Representative blots of mitochondrial OXPHOS complex subunits. The depicted data are the means ± SEM, n = 6/group. ^ρρ^*P* < 0.01/^ρ^*P* < 0.05 vs the HG group, ^ηη^*P* < 0.01/^η^*P* < 0.05 vs the HG + SIR group, ^θθ^*P* < 0.01/^θ^*P* < 0.05 vs the HG + SIR + Mel group. HG, high glucose. SIR, simulated ischemia reperfusion. Mel, melatonin. CC, Compound C.

As shown in Fig. 6a–d, melatonin markedly increased mitochondrial SOD activity and ATP production, decreased mitochondrial MDA and H_2_O_2_ generation (*P* < 0.05, compared with the HG + SIR group), while these effects were abolished by either Compound C or SIRT3 siRNA (*P* < 0.05, compared with the HG + SIR + Mel group). Additionally, western blot analysis showed that OXPHOS complexe I, II, III and IV were increased in melatonin-treated group (Fig. 6e, compared with the HG+SIR group. However, Compound C or SIRT3 siTNA also blunted this effect (Fig. 6e, compared with the HG + SIR + Mel group). These data suggested that melatonin could also attenuated mitochondrial oxidative stress and preserved mitochondrial function in high glucose cultured H9c2 cells. Importantly, AMPK and SIRT3 signaling mediated this action.

### Compound C and SIRT3 siRNA transfection reduced cellular SOD2, NRF1, TFAM expressions and increased cytosolic cytochrome c expression

**Figure 7:**
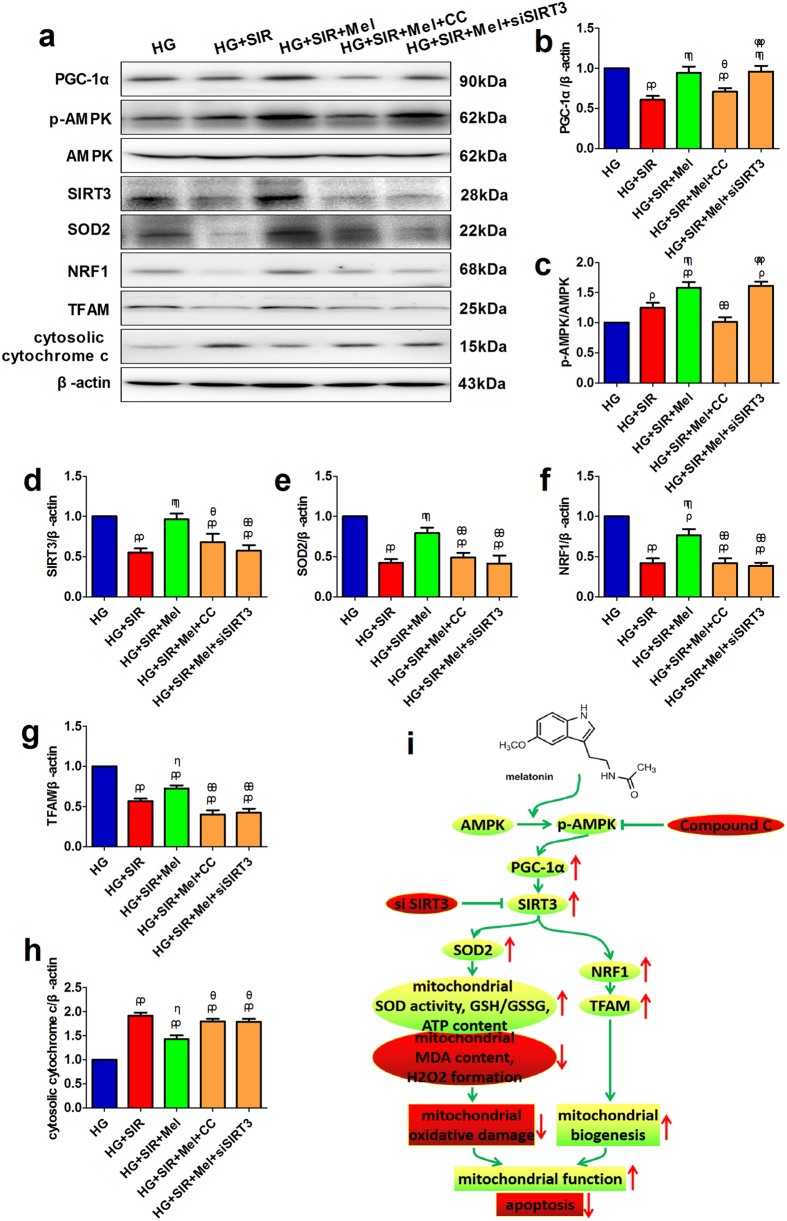
Compound C and SIRT3 siRNA transfection reduced cellular SOD2, NRF1, TFAM expressions and increased cytosolic cytochrome c expression. (**a**) Representative blots. (**b**) PGC-1α expression. (**c**) p-AMPK/AMPK ratio. (**d**) SIRT3 expression. (**e**) SOD2 expression. (**f**) NRF1 expression. (**g**) TFAM expression. (**h**) Cytosolic cytochrome c expression. (**i**) Proposed cardioprotective mechanisms by melatonin. The depicted data are the means ± SEM, n = 6/group. ^ρρ^*P* < 0.01/^ρ^*P* < 0.05 vs the HG group, ^ηη^*P* < 0.01/^η^*P* < 0.05 vs the HG + SIR group, ^θθ^*P* < 0.01/^θ^*P* < 0.05 vs the HG + SIR + Mel group, ^ψψ^*P* < 0.01/^ψ^*P* < 0.05 vs the HG + SIR + Mel + CC group. HG, high glucose. SIR, simulated ischemia reperfusion. Mel, melatonin. CC, Compound C.

Finally, we investigated the signaling molecules of AMPK-PGC-1α pathway, SIRT3 as well as mitochondrial biogenesis related protein expression. As shown in Fig. 7a–d, we found that melatonin significantly increased the p-AMPK/AMPK ratio and the expressions of PGC-1α and SIRT3 (*P* < 0.05, compared with the HG + SIR group). Compound C markedly reduced p-AMPK/AMPK ratio and also the expressions of PGC-1α and SIRT3 (*P* < 0.05, compared with the HG + SIR + Mel group), indicating that AMPK might be the up-stream signaling of PGC-1α and SIRT3. It is consistent with that SIRT3 siRNA did not significantly changed p-AMPK/AMPK ratio and PGC-1α expression (Fig. 7b,c, *P* > 0.05, compared with the HG + SIR + Mel group). Meanwhile, both SIRT3 siRNA and Compound C markedly inhibited the up-regulation of SOD2, NRF1 and TFAM induced by melatonin treatment (Fig. 7e–g, *P* < 0.05, compared with the HG + SIR + Mel group). Additionally, melatonin also significantly reduced cytosolic cytochrome c expression (Fig. 7h, *P* < 0.05, compared with the HG + SIR group), which was also blocked by Compound C or SIRT3 siRNA (*P* < 0.05, compared with the HG + SIR + Mel group). These results all indicated that the enhanced mitochondrial biogenesis and reduced oxidative stress were mediated by the activation of AMPK-PGC-1α-SIRT3 signaling (Fig. 7i).

## Discussion

There are several major findings in the present study. Firstly, we demonstrated that melatonin treatment is a potential strategy to ameliorate MI/R injury in type 1 diabetic state by enhancing mitochondrial biogenesis and preserving mitochondrial function. Secondly, AMPK-PGC-1α-SIRT3 signaling pathway was found to play a key role in melatonin’s cardioprotective action. To the best of our knowledge, this is the first study demonstrating the cardioprotective effect and potential mechanisms of melatonin against MI/R injury in type 1 diabetic state.

During recent years, the global prevalence of diabetes mellitus has grown sharply, which has become one of the most serious health issues worldwide^22^. Importantly, diabetes is associated with higher risk of cardiovascular complications such as coronary artery disease, congestive heart failure and acute myocardial infarction^23^. To make things worse, the long-term prognosis is also much worse than non-diabetic individuals with higher rates of residual ventricular dysfunction and overall mortality^23^,^24^. So far, the mechanisms have not been fully elucidated, but prolonged hyperglycemia-enhanced oxidative stress was indicated to be a crucial contributor^25^. Owing to its critical role in generating ATP and ROS, cardiomyocyte mitochondria has been specifically investigated. In diabetic myocardium, mitochondria is deemed as one of the major sources of free radicals. Numerous studies have reported that hyperglycemia markedly impaired mitochondrial morphology and function, causing electron leakage and O_2_^•−^ generation^25^,^26^. The enhanced oxidative stress has been confirmed to be a key contributor to increased myocardial vulnerablility to MI/R injury^25^. Additionally, reduced mitochondrial biogenesis was also found in diabetic myocardium. Since mitochondrial ATP synthesis is the main source of energy in the heart. Loss of mitochondrial number during MI/R injury can also result in cardiac contractile dysfunction^5^. In fact, plenty of studies have shown that rescuing mitochondria function by reducing mitochondria derived ROS and enhancing its biogenesis might be a promising therapeutic strategy against MI/R injury in diabetic state^5^,^27^. Consistent with these findings, we found that reperfusion injury significantly impaired cardiac function and enhanced myocardial apoptosis in diabetic animals. Meanwhile, MI/R injured rats also showed increased mitochondrial MDA, H_2_O_2_ generation and decreased mitochondrial SOD activity and ATP production, indicating that reperfusion injury also caused mitochondrial dysfunction.

As an endogenous circadian hormone, melatonin has been proved to be a promising antioxidant against MI/R injury due to its strong anti-oxidative capacity and high safety profile^9^,^28^,^29^,^30^,^31^. Previously, we and others also demonstrated that melatonin exerted a solid protective effect against MI/R injury in non-diabetic animals by activating multiple intracellular signaling pathways such as silent information regulator 1 (SIRT1), Janus kinase 2 (JAK2)/signal transducer and activator of transcription 3 (STAT3) and Notch1/Hairy and enhancer of split 1^29^,^32^,^33^. However, under type 1 diabetic state, its cardioprotective effect was poorly defined. Interestingly, a recent study by Bruno *et al*. showed that by activating cyclic AMP response element binding protein (CREB)-PGC-1α pathway, melatonin prevented mitochondrial dysfunction in rat skeletal muscle^13^. Moreover, Guo *et al*. demonstrated that melatonin pretreatment suppressed cadmium-induced hepatotoxicity by enhancing mitochondrial biogenesis^34^. We also demonstrated that in a non-diabetic MI/R rat model, melatonin treatment significantly reduced cardiac damage by reducing mitochondrial oxidative stress^32^. These studies all indicate that mitochondria may be a key target of melatonin in various pathological conditions. Consistent with these results, we found that under diabetic state, melatonin not only reduced mitochondrial oxidative stress markers and increased ATP production, but also significantly up-regulated NRF1 and TFAM expressions. Notably, TFAM is a downstream target of NRF1 that initiates the nuclear genes coding for subunits of the mitochondrial OXPHOS complex by specifically binding to the mitochondrial promoters^35^. As expected, we also observed that melatonin-treated group showed markedly enhanced mitochondrial OXPHOS complex subunits expression.

Another novel finding of this study is that we proved the importance of AMPK-PGC-1α-SIRT3 signaling in melatonin’s cardioprotective actions. Sirtuins are a family of class III histone deacetylases and their enzymatic activities are dependent on nicotinamide adenine dinucleotide^20^. So far, seven mammalian homologues of sirtuins (SIRT1-7) have been identified. These sirtuins are found to have distinct subcellular localizations and modulate various metabolic and stress-response signaling^20^. In cardiovascular system, SIRT1 and SIRT3 have been extensively studied. In our previous report, SIRT1 was demonstrated to be an important mediator of melatonin’s cardioprotective effect^4^,^33^. We found that in both diabetic and non-diabetic setting, melatonin suppressed MI/R-induced oxidative damage through activation of SIRT1 signaling. SIRT3 has been proved to be preferentially localized to mitochondria and control mitochondrial oxidative signaling and, consequently, the generation of ROS^20^,^36^. It has been demonstrated that SIRT3 knockdown in cells enhanced mitochondria ROS formation^37^ and cardiomyocytes of SIRT3 knockout mice exhibited markedly increased ROS generation^38^. Importantly, SIRT3 activation has also been demonstrated to reduce MI/R injury. Mohsen *et al*. demonstrated that losartan ameliorated MI/R injury by activating SIRT3 and thus enhancing thioredoxin-1 and catalase transcription^39^. Meanwhile, He *et al*. found that SIRT3 knock out impaired post-myocardial ischemia cardiac function by causing coronary microvascular dysfunction^40^. Notably, several studies also demonstrated the key role of SIRT3 in reducing myocardial injury in diabetic state. For example, SIRT3 knockout mice which were fed with 16 weeks of high-fat diet (HFD) exhibited increased myocardial ROS formation, impaired HIF signalling and reduced capillary density compared with HFD-fed wild type mice^41^. Also, Zeng *et al*. found that the SIRT3 expression in diabetic mice heart was markedly reduced^42^ while SIRT3 activation is the key mechanism of apelin-induced cardioprotective effect against myocardial injury in diabetic mice^43^. Consistently, we found that myocardial SIRT3 expression were significantly reduced after 1 month of STZ injection. Moreover, SIRT3 signaling was further impaired after the MI/R insult. Importantly, both our *in vivo* and *in vitro* data showed that melatonin significantly enhanced SIRT3 signaling and suppressed mitochondrial oxidative stress in diabetic cardiomyocytes. However, knockdown of SIRT3 inhibited the cytoprotective actions of melatonin on SIR-injured cardiomyoblasts in hyperglycemic state. These results indicated that reduced SIRT3 signaling might contribute to diabetic MI/R injury.

Park *et al*. showed that the SIRT3 activity in mouse liver and muscle was up-regulated by PGC-1α, suggesting that PGC-1α functions as an upstream regulator of SIRT3^44^. In fact, PGC1α-SIRT3 signaling has been demonstrated as essential for the regulation of mitochondrial oxidative stress and biogenesis^20^,^45^. Reduced PGC-1α expression have been linked to the pathogenesis of diabetes mellitus^46^. Meanwhile, PGC-1α has been reported to function as the critical downstream molecule of AMPK^47^,^48^,^49^. What is more, in the present experiment, we found AMPK-PGC-1α signaling was markedly down-regulated in diabetic myocardium. Therefore, we hypothesized that reduced AMPK-PGC-1α signaling resulted in decreased SIRT3 expression and melatonin might enhance SIRT3 signaling via the activation of AMPK-PGC-1α. As expected, our *in vivo* data showed that inhibition of AMPK significantly down-regulated PGC-1α and SIRT3 signaling. And the cellular experiment revealed that SIRT3 siRNA inhibited the up-regulation of SOD2, NRF1 and TFAM induced by melatonin treatment without affecting p-AMPK and PGC-1α protein levels. Therefore, we come to the conclusion that melatonin preserves mitochondrial function by reducing mitochondrial oxidative damage and enhancing its biogenesis, thus ameliorating MI/R injury in type 1 diabetic state. AMPK-PGC1α-SIRT3 axis plays an essential role in this process (Fig. 7i).

Previously, we and others demonstrated that melatonin membrane receptors mediated the cardiac protective actions of melatonin^28^,^33^. Moreover, we recently demonstrated that melatonin reduced flow shear stress (FSS)-induced bone marrow mesenchymal stem cell injury by activating melatonin membrane receptors and AMPK signaling^50^. Kunst *et al*. also reported that PGC-1α was the target genes of melatonin in murine retina cells. Additionally, melatonin membrane receptors participated in this action^51^. However, the potential role of melatonin membrane receptors in this study was unknown. In diabetic myocardium, whether melatonin modulates AMPK-PGC1α-SIRT3 signaling pathway via its membrane receptors and the detailed mechanisms remain further study.

Taken together, this study investigated the potential cardioprotective effect of melatonin against MI/R injury in diabetic state. We found that melatonin treatment effectively enhanced mitochondrial biogenesis and preserved mitochondrial function, thus reducing MI/R injury in type 1 diabetic state. More importantly, we demonstrated the critical role of AMPK-PGC-1α-SIRT3 signaling pathway in melatonin’s cardioprotective actions. These results showed that melatonin treatment might be a promising therapeutic strategy against MI/R injury in diabetic state. Further studies using SIRT3 deficient animals are warranted to confirm these findings.

## Methods

### Ethics Statement and Aniamls

Eight-week-old male Sprague-Dawley (SD) rats (weighing 180–220 g) were obtained from the General Hospital of Shenyang Military Area Command Experimental Animal Center and kept in adequate-size cages with standard rat chow and water *ad libitum*. The room was kept at constant temperature and humidity with a light/dark cycle (12/12 h). The animal care and experimental procedures of this study were approved by the General Hospital of Shenyang Military Area Command Committee on Animal Care. All experiments were carried out in accordance with the Guide for the Care and Use of Laboratory Animals published by the US National Institutes of Health (NIH Publication, 8th Edition, 2011).

### Establishment of type 1 diabetic model

Type 1 diabetic model was induced as described previously^52^. Briefly, the SD rats were fasted overnight and received streptozotocin (STZ, Sigma-Aldrich, MO, USA, 50 mg/kg/d, i.p., dissolved in 0.1 mol/l citrate buffer, pH 4.5) injection for 3 consecutive days. 7 days after the injection, the rats with fasting plasma glucose (PG) above 11.1 mmol/l were classified as diabetic. Intraperitoneal glucose tolerance test (IPGTT) and oral glucose tolerance test (OGTT) were performed by administering glucose (2 g/kg) by gastric lavage or intraperitoneal injection to further confirm the diabetic model^4^.

### Myocardial ischemia/reperfusion model and experimental design

MI/R rat model was established as described previously^53^. The rats were anesthetized with 1% sodium pentobarbital (40 mg/kg). Myocardial ischemia surgery was performed by exposing the heart with a left thoracic incision. A silk ligature was placed around left anterior descending coronary artery. After 30 min of ischemia, the ligature was released and the myocardium was reperfused for 3 hours. Sham-operated group were subjected to the same procedures except that the ligature was left untied. During the whole ischemia and reperfusion period, the left ventricular function was measured by a hemodynamic monitoring system (Chengdu Taimeng technology Co., Ltd., China). Left ventricular systolic pressure (LVSP), first derivative of left ventricular pressure (+dP/dt_max_ and −dP/dt_max_) were directly obtained by computer algorithms at baseline, ischemia for 30 min and reperfusion for 1, 2, and 3 hours.

The animals were randomly assigned to the following groups, namely, (1) T1D + Sham group; (2) T1D + MI/R + vehicle (V) group; (3) T1D + MI/R + Mel group; (4) T1D + MI/R + Mel + Compound C (CC) group. Melatonin (10 mg/kg, diluted in sterile saline containing 0.5% ethanol) was orally administered for 5 days before the surgery and intraperitoneally injected once 10 min before the reperfusion. Compound C (Sigma-Aldrich, MO, USA, 0.25 mg/kg) was intravenously infused 15 min before the reperfusion.

### Determination of myocardial infarct size and apoptosis

Myocardial infarct size were measured after 6 h of reperfusion by Evans blue/TTC double-staining technique (Solarbio Technology, Beijing, China) as described previously^54^. The area at risk (AAR), infarct size (INF) and viable area were assessed digitally using Image-Pro Plus software (Media Cybernetics, MA, USA). The infarct size was expressed as a percentage of infarct area (INF) over total AAR (INF/AAR × 100%).

Myocardial apoptosis was measured with an *in situ* cell death detection kit (Roche Molecular Biochemicals, Mannheim, Germany) as previously described^55^. The apoptotic index was expressed as the number of apoptotic nuclei/the total number of nuclei counted × 100%.

### Immunohistochemistry analysis

Immunohistochemistry staining was performed as previously described^56^. The cardiac tissue was fixed with 10% formalin and embedded in paraffin. Anti-PGC-1α antibody (Cell Signaling Technology, MA, USA, 1:100 dilution), anti-SIRT3 antibody and anti-SOD2 antibody (Santa Cruz, CA, USA, 1:100 dilution) were used as the primary antibody. The positive area was detected by 3,3′-Diaminobenzidine (DAB) staining (Zhongshan biotechnology, Beijing, China). IgG antibody was used as a negative control. The images were photographed at 200× magnification (Olympus BX-63, JAPAN).

### Cell treatment and *in vitro* experimental protocol

H9c2 cardiomyoblast cells (Tiancheng Technology, Shanghai, China) were cultured as described previously^53^. Simulated ischemia/reperfusion treatment was carried out as described by our previous study^53^. Briefly, the cells were initially incubated with the ischemic buffer [0.49 MgCl_2_, 0.9 CaCl_2_, 12 KCl, 137 NaCl, 4 HEPES, 0.75 sodium dithionate, 10 deoxyglucose and 20 lactate (in mmol/L)] in a normal cell culture incubator. After 2 hours of simulated ischemia, the cells were returned to normal culture medium for 4 hours of simulated reperfusion. Before and after the SIR treatment, the cells received different treatment.

As previously described^28^,^29^, H9c2 cells were incubated in high-glucose medium (33 mmol/l) for 6 hours before the SIR treatment and during the entire reperfusion period (4 hours) to mimic the *in vivo* diabetic setting. Normal medium (containing 5.5 mmol/l glucose) was used as a control. HG-treatment was co-administered with or without melatonin (10 μmol/l) to evaluate its cytoprotective effect. Compound C (3 μmol/l) was administered for 6 hours before the SIR exposure to inhibit the AMPK signaling.

### Small interfering RNA transfection

SIRT3 siRNA duplex solution, transfection reagent and medium were all obtained from Santa Cruz biotechnology (CA, USA). The transfection of SIRT3 and control siRNA was carried out in accordance with the manufacture’s instruction as described previously^28^. The knockdown capacity of SIRT3 siRNA was evaluated by western blot analysis.

### Cellular viability assessment

Cellular viability was evaluated by 3-(4,5-dimethylthiazol-2-yl)-2,5-diphenyltetrazolium bromide (MTT) assay kit (Solarbio Technology, Beijing, China). The results was read using a microtiter plate reader (SpectraMax 190, Molecular Device, USA) and calculated by dividing the optical density of samples by that of the control group^55^.

### Isolation of myocardial mitochondria and cytosol fraction

The isolation of myocardial mitochondrial was performed strictly as described by our previous study^32^. In brief, the cardiac tissue or cells was washed and homogenized using buffer A [0.3 phenylmethylsulfonyl fluoride, 1 orthovanadate, 1 NaF, 1 EDTA, 10 Tris-HCl, 250 sucrose (in mmol/l), added with 5 μg/ml each of leupeptin, aprotinin, and pepstatin A], followed by serial centrifugations (1000, 10000 and 100000 g). The 100000 g supernatant was collected and defined as the cytosolic fraction, while the 10000 g pellet was washed, collected and resuspended in buffer B [0.3 PMSF, 1 NaF, 1 orthovanadate, 10 EDTA, 20 Tris-HCl, 150 NaCl (in mmol/l), 1% NP-40, added with 0.5 lg/mL pepstatin A and 5 lg/mL each of leupeptin and aprotinin], followed by 10 min of centrifugation (21000 g). Then, the supernatant was carefully collected and defined as the mitochondrial fraction.

### Mitochondrial oxidative stress and functional evaluation

The mitochondrial oxidative stress markers (SOD activity, MDA content, H_2_O_2_ formation) and ATP production was measured using commercially available kits (Beyotime biotechnology, Shanghai, China) as described previously^32^. Additionally, we also measured the cytosolic cytochrome c expression to determine the mitochondrial function and apoptosis.

### Western blot analysis

Western blot analysis was carried out as described previously^30^. In brief, proteins of myocardial tissue and H9c2 cardiomyoblasts were prepared and separated on SDS-PAGE gels. Then, they were transferred to polyvinylidene difluoride membrane (Millipore, USA) and incubated overnight (4 °C) with p-AMPK, AMPK, PGC-1α, Bcl-2, Bax, caspase-3 and cleaved caspase-3 antibodies (Cell Signaling Technology, MA, USA, 1:1000 dilution), SIRT3, SOD2, NRF1, TFAM, cytochrome c and β-actin antibodies (Santa Cruz, CA, USA, 1:500 dilution) and total OXPHOS antibody cocktail (Abcam biotechnology, Cambridge, UK, 1:1000 dilution). Then, the membranes were washed and probed with the secondary antibodies for 2 hours (37 °C). Finally, the positive bands were scanned with ChemiDocXRS and analyzed with Imagelab software system (Bio-Rad, CA, USA).

### Statistical analysis

Values are presented as mean ± SEM and were subjected to a 2-tailed Student’s t test between two groups or by ANOVA followed by post-hoc Bonferroni’s multiple comparison tests (GraphPad Prism software version 5.0, GraphPad Software, CA, USA). Any *P* value < 0.05 was considered statistically significant.

## Additional Information

**How to cite this article:** Yu, L. *et al*. Melatonin ameliorates myocardial ischemia/reperfusion injury in type 1 diabetic rats by preserving mitochondrial function: role of AMPK-PGC-1α-SIRT3 signaling. *Sci. Rep.* **7**, 41337; doi: 10.1038/srep41337 (2017).

**Publisher's note:** Springer Nature remains neutral with regard to jurisdictional claims in published maps and institutional affiliations.

## Supplementary Material

Supplementary Files
