# Supplementary material for: Melatonin ameliorates myocardial ischemia/reperfusion injury in type 1 diabetic rats by preserving mitochondrial function: role of AMPK-PGC-1α-SIRT3 signaling
Source: Sci Rep. 2017 Jan 25;7:41337. doi: 10.1038/srep41337 (PMC5264601; doi:10.1038/srep41337)

Supplementary Information

Title of manuscript:

Melatonin ameliorates myocardial ischemia/reperfusion injury in type 1 diabetic rats by preserving mitochondrial function: role of AMPK-PGC-1α-SIRT3 signaling

Authors:

Liming Yu, Bing Gong, Weixun Duan, Chongxi Fan, Jian Zhang, Zhi Li, Xiaodong Xue, Yinli Xu, Dandan Meng, Buying Li, Meng Zhang, Bin Zhang, Zhenxiao Jin, Shiqiang Yu, Yang Yang, Huishan Wang

Supplementary information includes:

Supplementary Fig. S1, S2 and Supplementary Fig. 1-7 (The full length blots in Fig. 1-7)

Supplementary Fig. S1: High glucose incubation impaired AMPK/PGC-1α signaling in H9c2 cells. (**a**) Representative blots. Cellular p-AMPK, PGC-1α, SIRT3 and SOD2 expressions were evaluated after 8 hours of HG incubation (25mmol/l, 33mmol/l). Normal glucose medium (containing 5.5mmol/l glucose) was used as a control. Mannitol was used to rebalance the glucose concentration among the experimental groups. (**b**) p-AMPK/AMPK ratio. (**c**) PGC-1α expression. (**d**) SIRT3 expression. (**e**) SOD2 expression. The depicted data are the means ± SEM, n= 6/group. $^{@@}P < 0.01$/$^{@}P < 0.05$ vs the NG group, $^{\&\&}P < 0.01$/$^{\&}P < 0.05$ vs the NG+mannitol group, $^{\wedge\wedge}P < 0.01$/$^{\wedge}P < 0.05$ vs the HG 25mM group. NG, normal glucose. HG, high glucose.

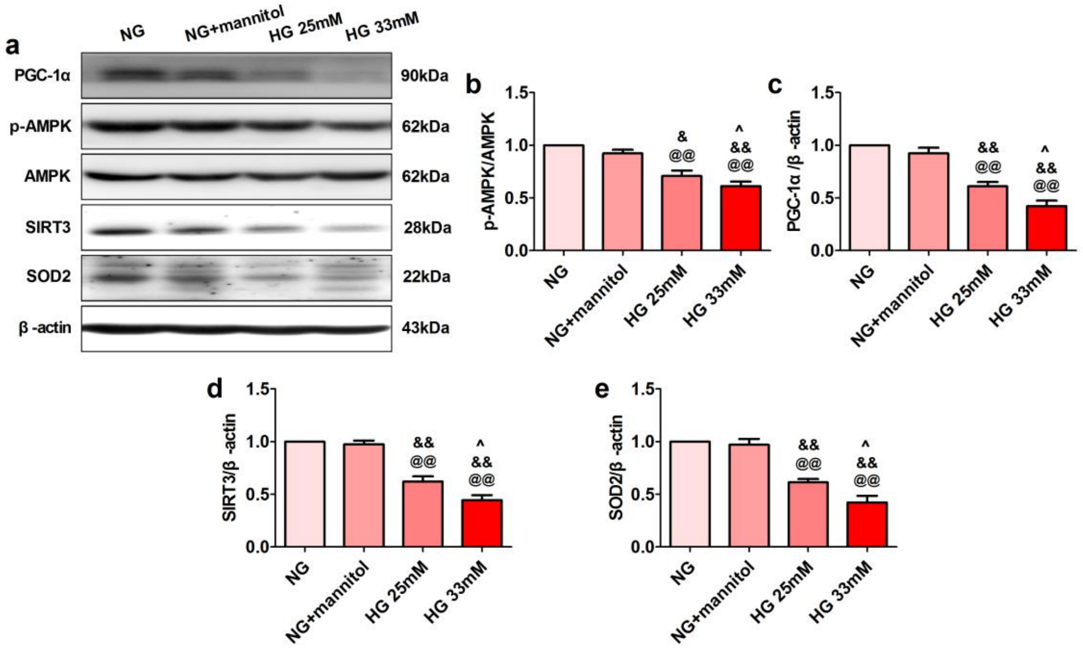

Supplementary Fig. S2: Evaluation of the knockdown capacity of SIRT3 siRNA on SIR-treated H9c2 cells and the effect of Compound C on diabetic rats.

Myocardial ischemia/reperfusion surgery was performed after 1 month of streptozotocin injection. Compound C (0.25mg/kg) or vehicle (sterile saline containing 0.1% DMSO) was intravenously infused 15 min before the reperfusion. (**a**) Left ventricular systolic pressure. (**b**) and (**c**) The first derivative of left ventricular pressure (+dP/dt_{max} and -dP/dt_{max}). Cardiac functional data was continuously monitored during the ischemia (30 min) and reperfusion period (3 hours). (**d**) Representative blots. (**e**) Caspase-3 expression. (**f**) Cleaved caspase-3 expression. (**g**) Knockdown capacity of SIRT3 siRNA. The depicted data are the means ± SEM, n= 6/group.$^{\pi\pi}P < 0.01$ vs the SIR group, $^{\sigma\sigma}P < 0.01$ vs the SIR+control siRNA group. T1D, type 1 diabetes. V, vehicle. CC, Compound C. MI/R, myocardial ischemia/reperfusion. SIR, simulated ischemia reperfusion.

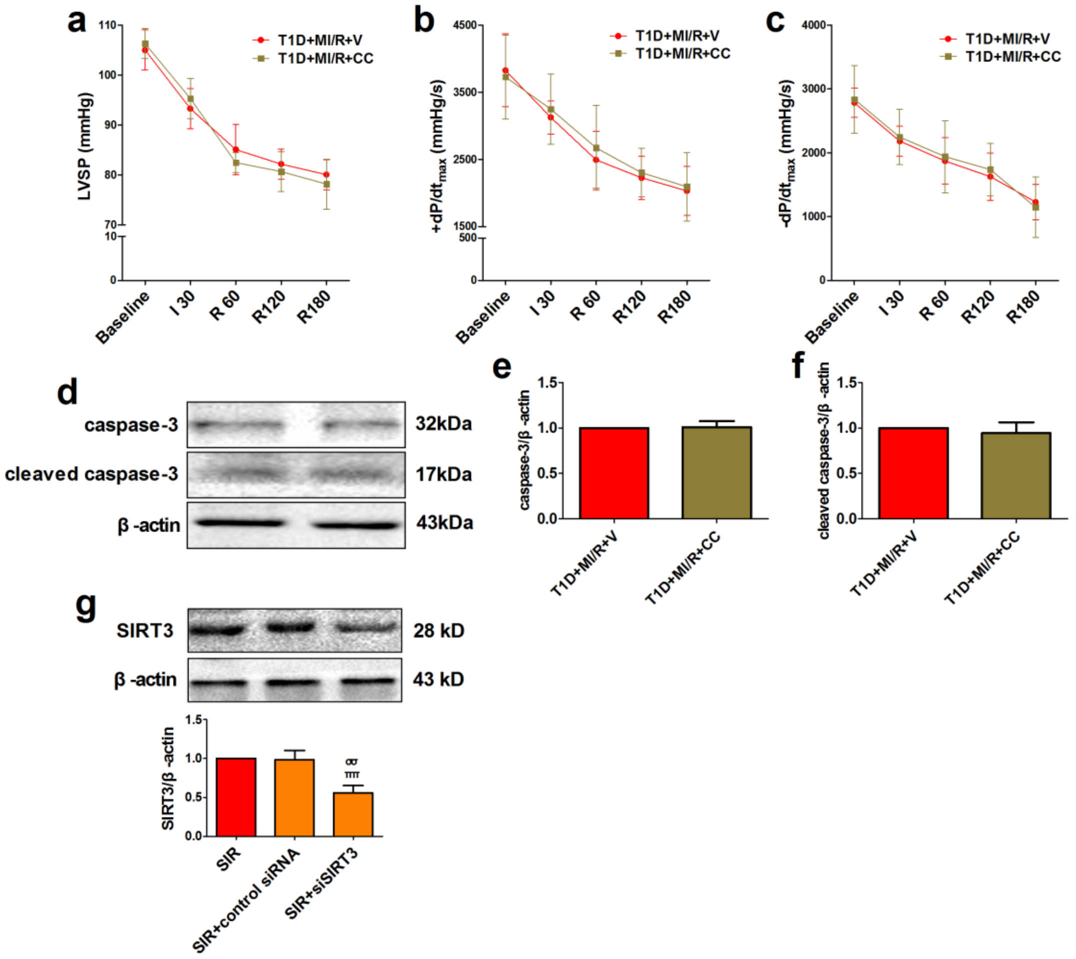
a
LVSP (mmHg)
T1D+MI/R+V
T1D+MI/R+CC
Baseline
I 30
R 60
R120
R180
b
+dP/dtmax (mmHg/s)
c
-dP/dtmax (mmHg/s)
d
caspase-3
cleaved caspase-3
β -actin
32kDa
17kDa
43kDa
e
caspase-3/β -actin
f
cleaved caspase-3/β -actin
g
SIRT3
β -actin
28 kD
43 kD
SIRT3/β -actin
SIR
SIR+control siRNA
SIR+siSIRT3

Supplementary Fig. 1 The full length blots in Fig. 1

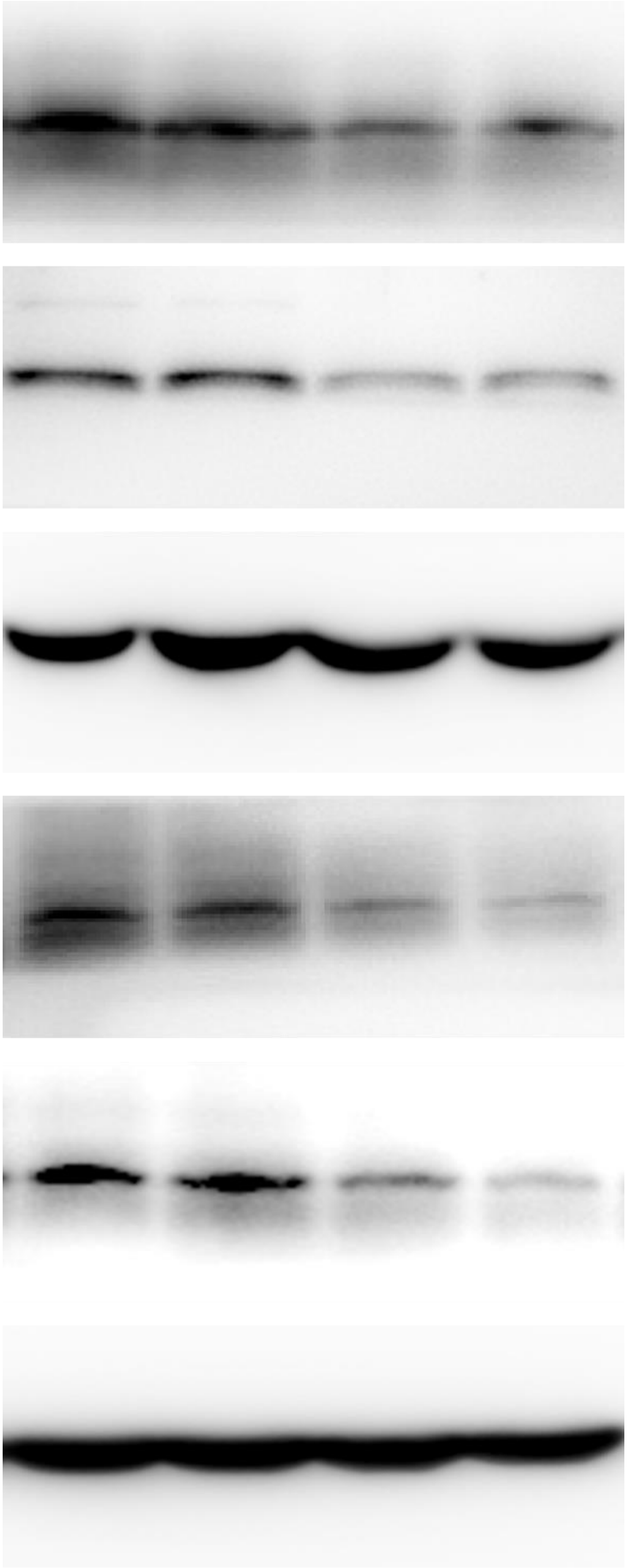

Supplementary Fig. 2 The full length blots in Fig. 2

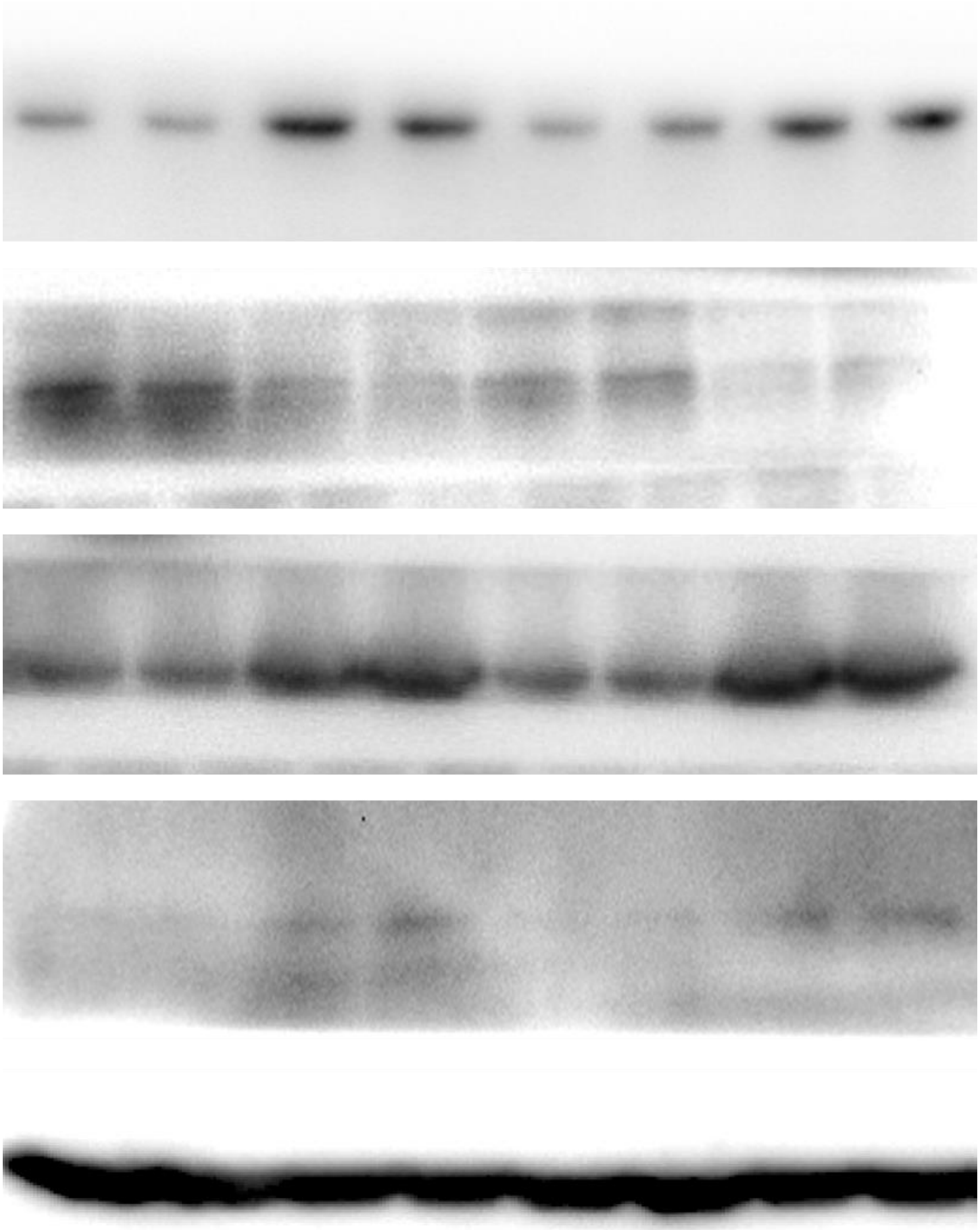

Supplementary Fig. 3 The full length blots in Fig. 3

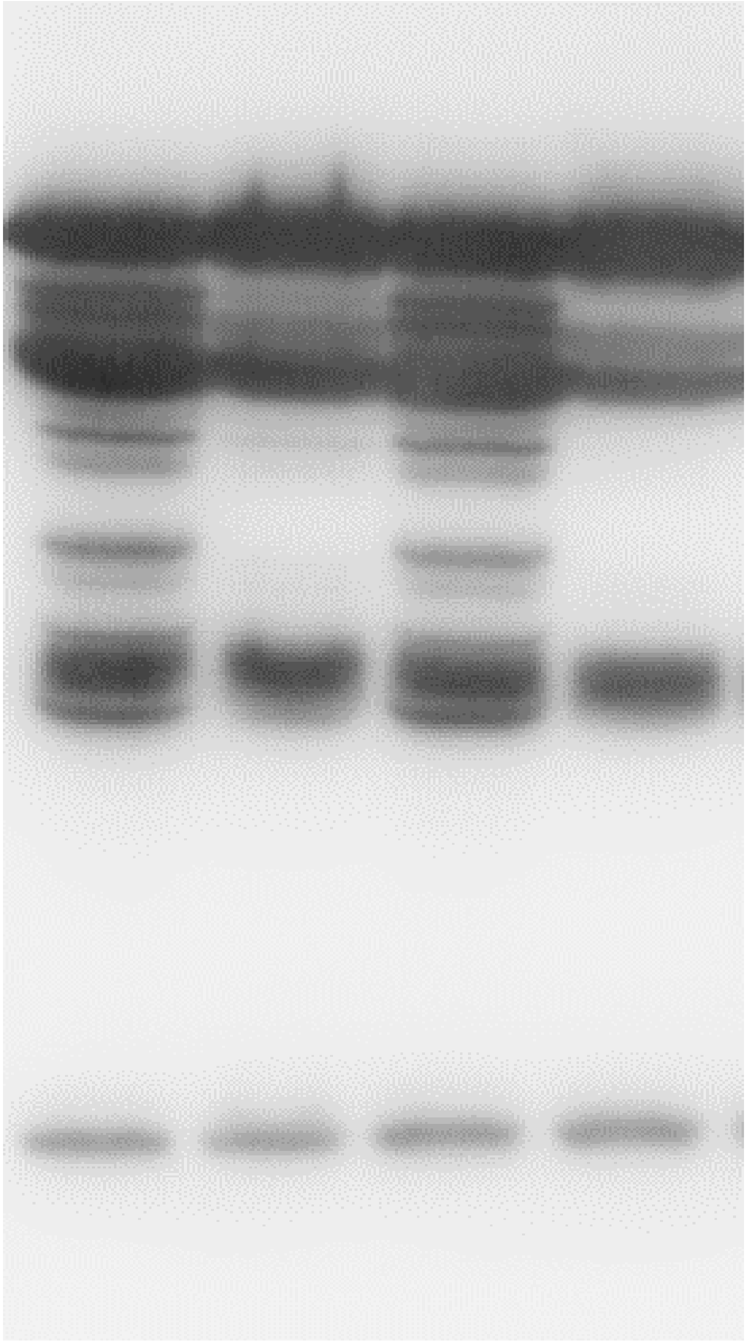

Supplementary Fig. 4 The full length blots in Fig. 4

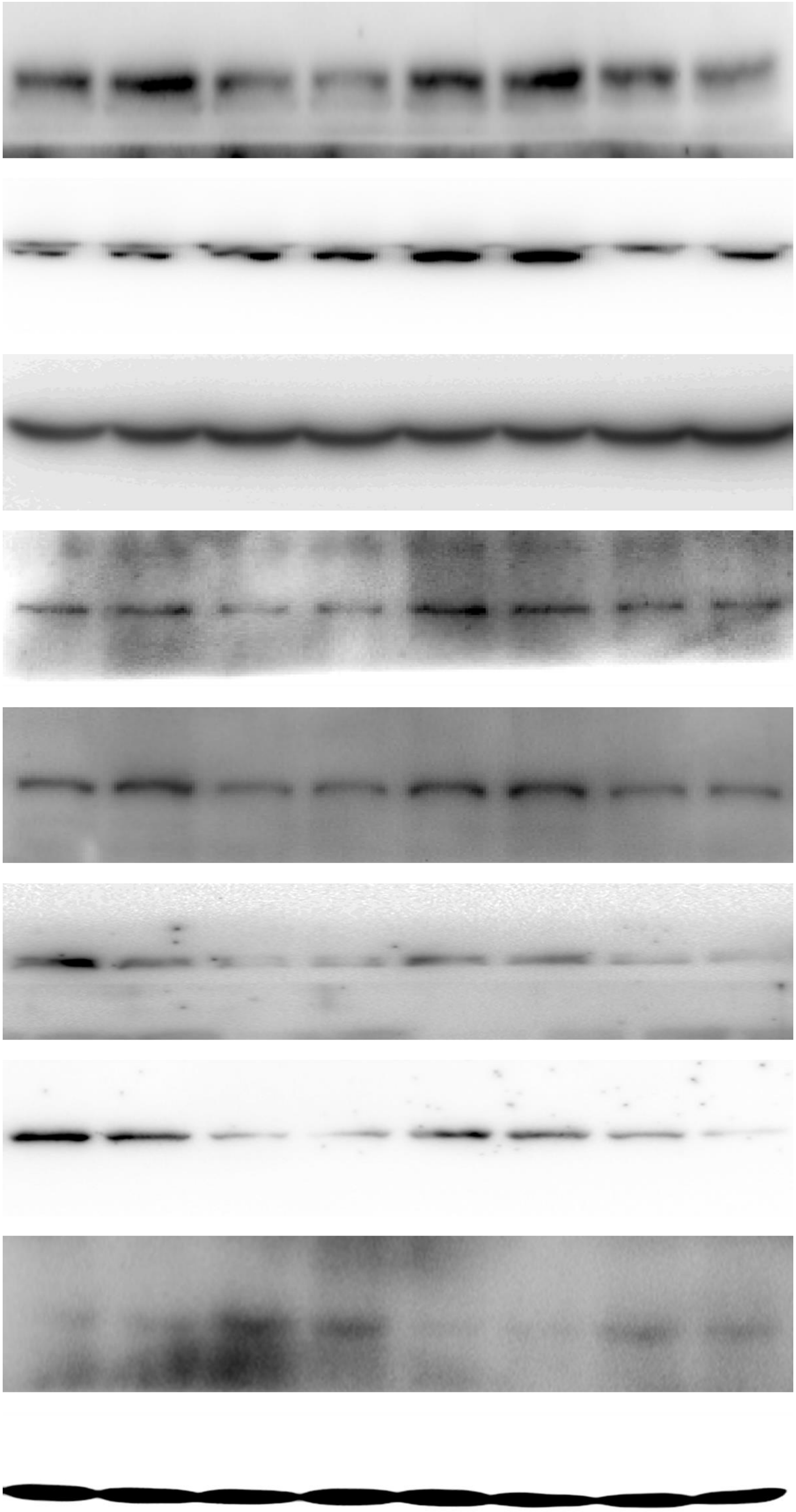

Supplementary Fig. 5 The full length blots in Fig. 5

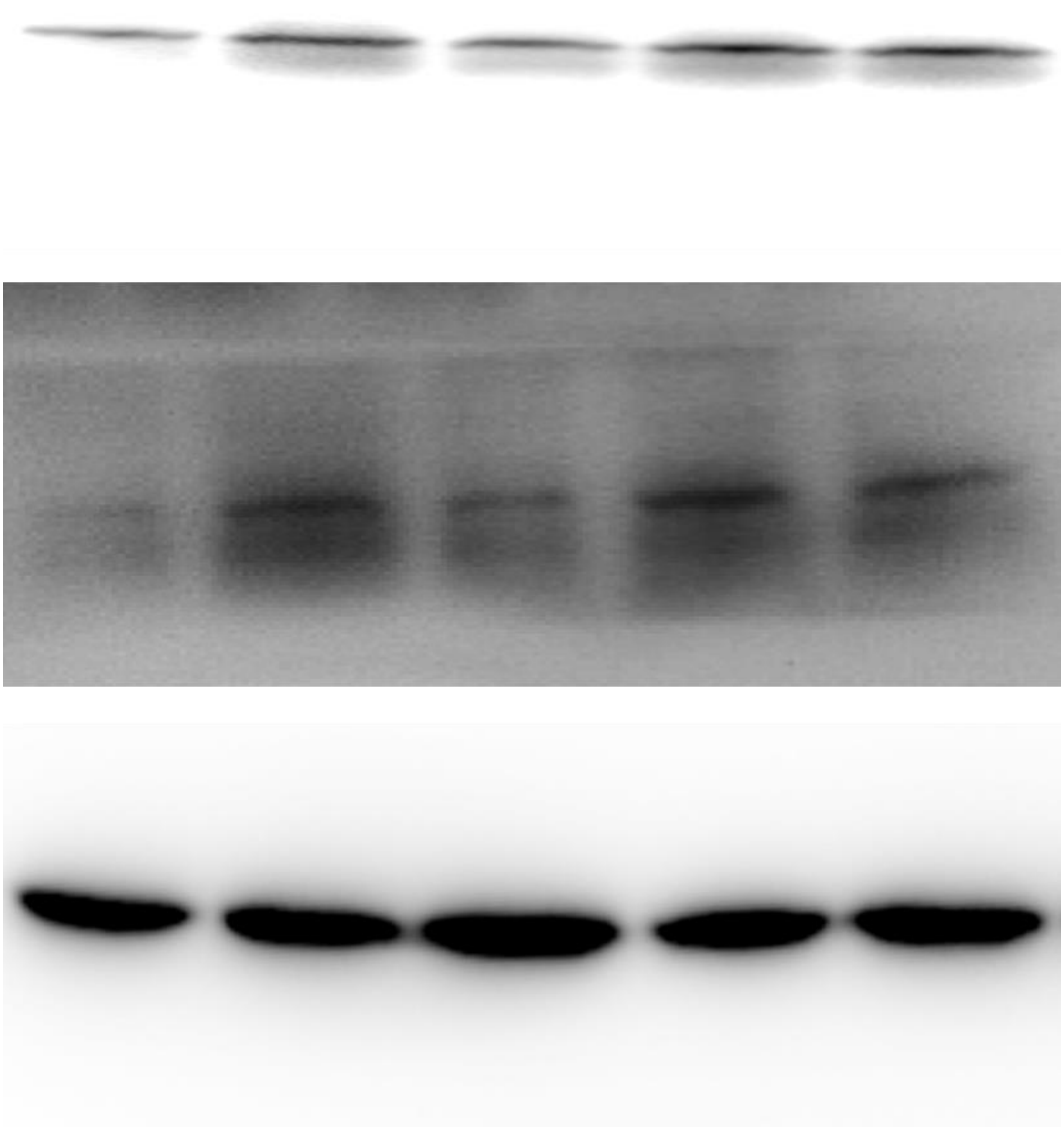

Supplementary Fig. 6 The full length blots in Fig. 6

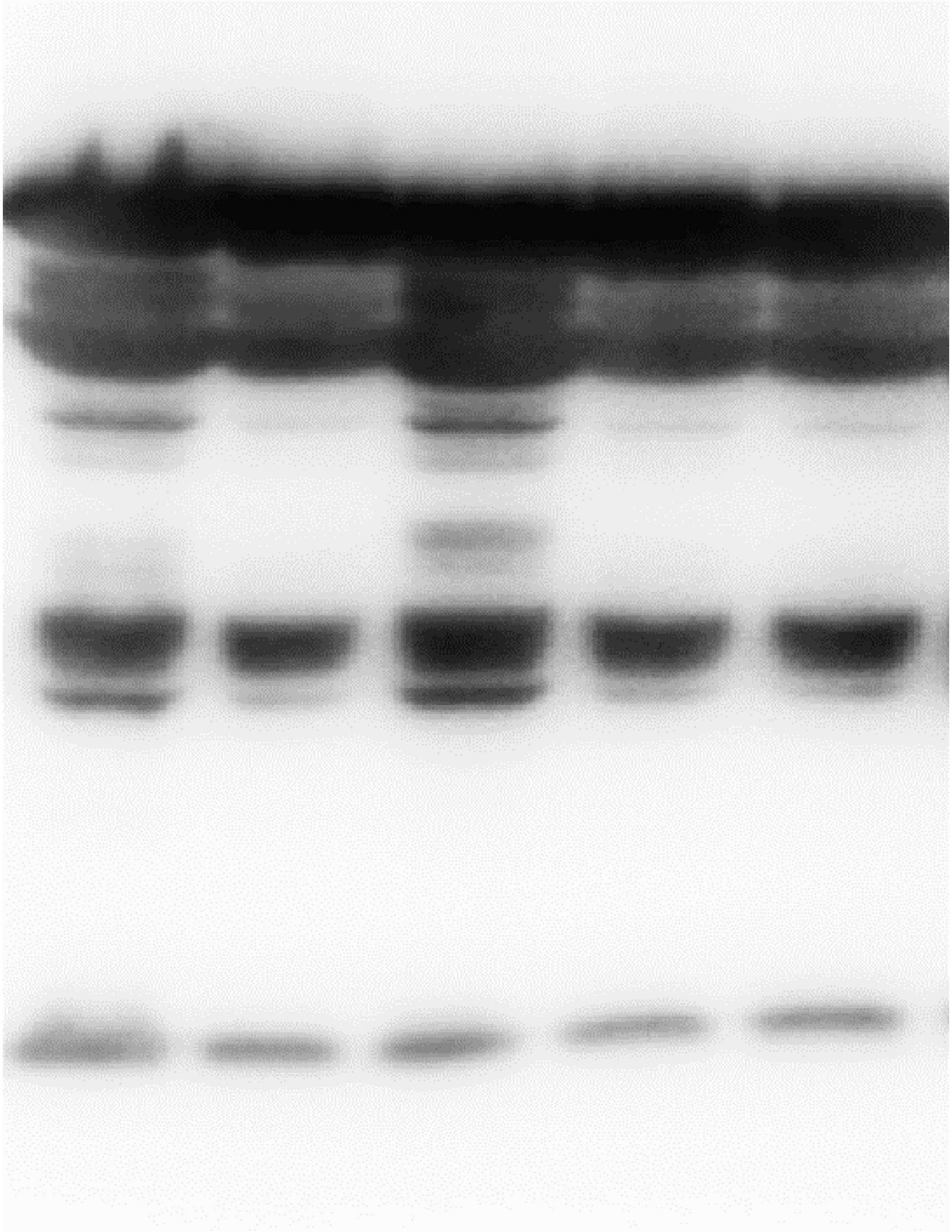

Supplementary Fig. 7 The full length blots in Fig. 7

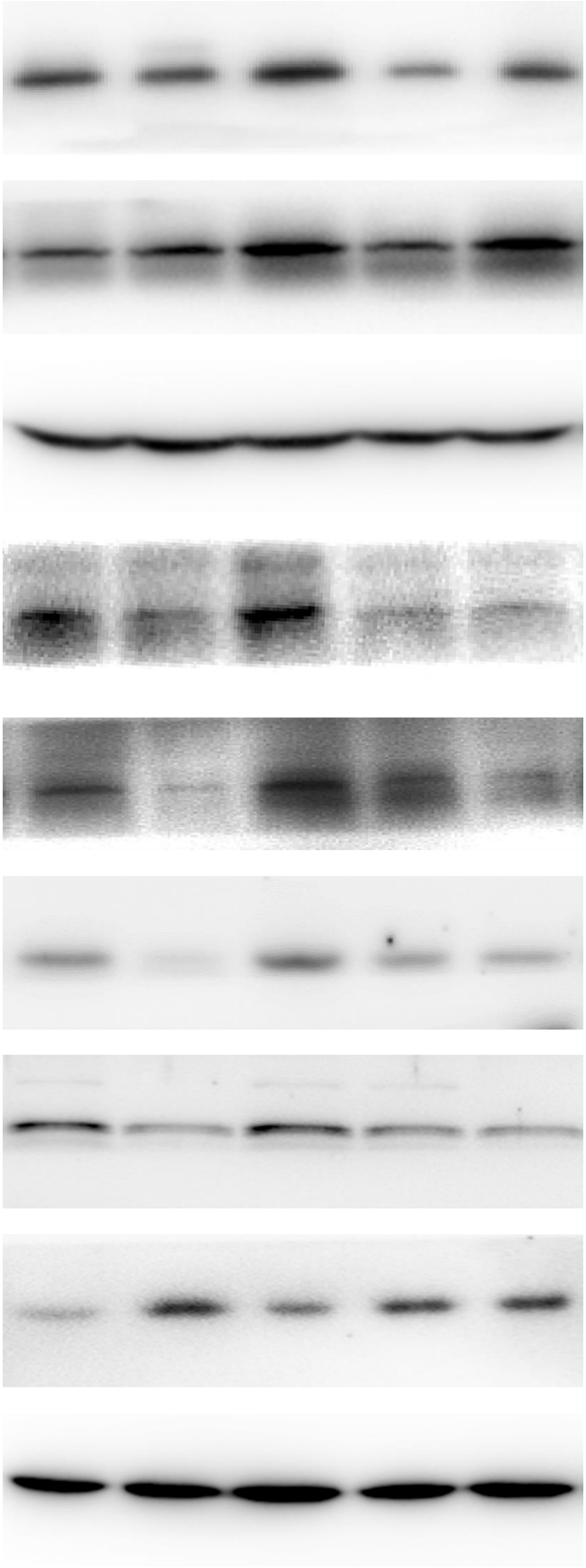

Supplement: Supplementary Files [file srep41337-s1.pdf]
